# Network Pharmacology and Experimental Validation to Reveal Effects and Mechanisms of Icariin Combined with Nobiletin against Chronic Obstructive Pulmonary Diseases

**DOI:** 10.1155/2022/4838650

**Published:** 2022-11-03

**Authors:** Ruilong Lu, Kexin Xu, Yanqin Qin, Xuejie Shao, Miaomiao Yan, Yixi Liao, Bo Wang, Jie Zhao, Jiansheng Li, Yange Tian

**Affiliations:** ^1^Co-Construction Collaborative Innovation Center for Chinese Medicine and Respiratory Diseases By Henan & Education Ministry of PR, Henan University of Chinese Medicine, Zhengzhou 450046, Henan, China; ^2^Henan Key Laboratory of Chinese Medicine for Respiratory Disease, Henan University of Chinese Medicine, Zhengzhou 450046, Henan, China; ^3^Institute for Respiratory Diseases, The First Affiliated Hospital, Henan University of Traditional Chinese Medicine, Zhengzhou 450008, Henan, China

## Abstract

**Background:**

Chronic obstructive pulmonary disease (COPD) is a long-term respiratory disorder marked by restricted airflow and persistent respiratory symptoms. According to previous studies, icariin combined with nobiletin (I&N) significantly ameliorates COPD, but the therapeutic mechanisms remain unclear.

**Purpose:**

The aim of the study is to investigate the therapeutic mechanisms of I&N against COPD using network pharmacology and experimental validation.

**Methods:**

The targets of I&N and related genes of COPD were screened and their intersection was selected. Next, the protein-protein interaction (PPI) networks, Gene Ontology (GO) and Kyoto Encyclopedia of Genes and Genomes (KEGG) pathway enrichment analyses were performed. Further, a COPD rat model was established to validate the effect and mechanisms of I&N.

**Results:**

445 potential targets I&N were obtained from SwissTargetPrediction, STITCH 5.0, and PharmMapper databases. 1831 related genes of COPD were obtained from GeneCards, DrugBank, and DisGeNet databases. 189 related genes were screened via matching COPD targets with I&N. 16 highest score targets among 189 targets were obtained according to PPI networks. GO and KEGG pathway enrichment analyses of 16 highest score targets suggested that these key genes of I&N were mostly enriched in the tumor necrosis factor (TNF) pathway, mitogen-activated protein kinase (MAPK) pathway, and phosphatidyl inositol 3-kinase (PI3K)-protein kinase B (AKT) pathway. Therefore, the treatments of I&N for COPD were connected with inflammation-related pathways. In in vivo experiments, the studies indicated that I&N improved the lung function and alleviated the damage of pulmonary histopathology. Moreover, I&N reduced levels of interleukin (IL)-6, IL-1*β*, and TNF-*α* in lung tissues of COPD rats and inhibited the activation of the MAPK pathway and PI3K-Akt pathway.

**Conclusions:**

Icariin combined with nobiletin has therapeutic effects on COPD by inhibiting inflammation. The potential mechanisms of I&N may relate to the MAPK pathway and PI3K-Akt pathway.

## 1. Introduction

Chronic obstructive pulmonary disease is the most common disease of the respiratory system with high morbidity and mortality and endangers public health [1]. Lung and systemic inflammation and lung injury are the main pathophysiology changes in COPD [2]. Nowadays, various treatment strategies are available for COPD, including bronchodilators and anti-inflammatory agents, and bronchodilator therapy is the most common treatment against COPD [2]. However, serious side effects, such as potentially paradoxical bronchospasm, may arise due to adhibition of bronchodilator therapy [3]. Traditional Chinese medicine (TCM) has special superiorities for treating COPD. Bufei Yishen formula (BYF), which is an effective therapeutic strategy for COPD, exerts various positive effects for COPD patients via inhibition of inflammation [4]. Icariin and nobiletin, two active ingredients screened from BYF, have been reported to have anti-inflammatory, antiapoptosis, and antioxidant effects against several inflammatory diseases [5–7]. The effects on improving the lung function and inhibiting the inflammatory response of I&N in COPD rats had been proved in previous studies. However, the mechanisms of I&N for treatment of COPD remain unclear and the traditional experimental approaches are difficult to elucidate the mechanisms and key action targets of I&N for COPD.

Network pharmacology is a strategy based on multidirectional pharmacology, system biology, network analysis, and computational biology, which systematically expounds the potential targets and mechanisms of TCM [8]. In this method, the relationship networks of herb, compound, target, pathway, and disease are established, which reveal the molecular basis and forecast the pharmacological mechanisms [9].

**Figure 1:**
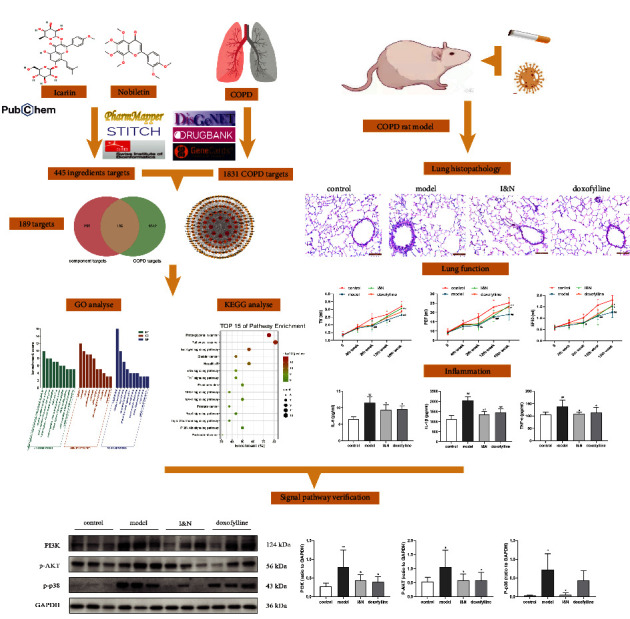
Schematic flowchart.

In this study, the targets of I&N and related genes of COPD were screened and the ingredients-disease targets network was established. Then, the potential molecular mechanisms were revealed via gene enrichment analysis and molecular docking. Finally, the COPD rat model was established to verify therapeutic effects and potential pathway of I&N against COPD (Figure 1). Therefore, the primary goals of this study were (1) to screen related genes of COPD and the potential targets of I&N; (2) to dissect the underlying mechanisms of I&N for COPD using network pharmacology; and (3) to validate anti-inflammatory effects and the potential pathway of I&N for treatment of COPD.

## 2. Materials and Methods

### 2.1. Network Pharmacology

#### 2.1.1. Screening the Molecular Targets of Icariin and Nobiletin

The canonical SMILES of icariin and nobiletin were acquired by searching the keywords of “icariin” (Compound CID: 5318997) and “nobiletin” (Compound CID: 72344) from PubChem [10]. The molecular targets of icariin and nobiletin were filtered by searching the canonical SMILES of icariin and nobiletin from SwissTargetPrediction (https://www.swisstargetprediction.ch/), STITCH 5.0 (https://stitch.embl.de/) [11], and PharmMapper (https://lilab-ecust.cn/pharmmapper/) [12].

#### 2.1.2. Acquisition of Gene Targets for COPD

The related genes of COPD were screened via the keywords of “chronic obstructive pulmonary disease” in GeneCards (https://www.genecards.org/) [13], DrugBank (https://go.drugbank.com/) [14], and DisGeNet (https://www.disgenet.org/) [15]. Then, all targets of components and COPD were submitted to UniProtKB (https://www.uniprot.org/) [16] to acquire the standardized gene symbols.

#### 2.1.3. PPI Network Construction

First, we intersected the obtained components targets with the genes associated with COPD and obtained a Venn diagram of the intersected gene symbols. Then, a PPI network was built using STRING [17] and Cytoscape 3.8.2. To screen the key targets, the topological characteristics were analyzed of the PPI network. First, the gene symbols were chosen by the degree score. Next, the betweenness centrality (BC), closeness centrality (CC), degree, and average shortest path length (ASPL) were calculated by Cytoscape to indicate the potential targets.

#### 2.1.4. Enrichment of GO and KEGG Pathways

The GO and KEGG pathways enrichments of the topological potential targets were analyzed in DAVID 6.8 [18]. The *p* value <0.05 was set as a significant difference for KEGG pathway analysis.

#### 2.1.5. Molecular Docking

The 3D structures of icariin and nobiletin were acquired from PubChem and were transformed from their original constructions into PDB formats using Open Babel 3.1.1. From RCSB Protein Data Bank, the X-ray crystal structures of key proteins were obtained [19]. Seven protein targets were studied: AKT1 (PDB ID: 2UZR), TNF (PDB ID: 7KP9), VEGFA, (PDB ID: 7LL8), EGFR, (PDB ID: 5Y9T), JUN, (PDB ID: 5T01), MMP9, (PDB ID: 1L6J), and SRC, (PDB ID: 2BDF). The water molecules were deleted and hydrogen atoms were added in optimizer of structures using AutoDock Tool 1.5.6. Then, the receptor proteins were docked with ligand molecules via AutoDock. All of options were default setting for docking run. Finally, the molecular docking results were visualized by PyMoL 2.2.3, which acquire the highest scores.

### 2.2. Experiment Validation

#### 2.2.1. Chemicals and Reagents

Sprague-Dawley (SD) rats were purchased from Beijing Vital River Laboratory Animal Technology Co., Ltd (220 ± 20 g, No.110011211105823815, Beijing, China). Hongqi Canal® Filter tip cigarette was purchased from Henan Tobacco Industry (Zhengzhou, China). *Klebsiella pneumoniae* (46117-5a1) was purchased from National Center for Medical Culture Collections (Beijing China). Icariin (Cas, 489-32-7) and nobiletin (Cas, 478-01-3) were purchased from Chengdu Must Bio-Technology (Chengdu, China). Doxofylline was obtained from Heilongjiang Fuhe Pharmaceutical Group Co., LTD. (Heilongjiang, China). The rat ELISA kits of IL-6 (Cat.No.550319) were purchased from BD Biosciences (California, America). The rat ELISA kits of IL-1*β* (E-EL-R0012c) and TNF-*α* (E-EL-R2856c) were purchased from Elabscience Biotechnology Co., Ltd (Wuhan, China). The antibodies for rat of PI3K (GTX55747, Gene Text) and P-AKT (GTX128414, Gene Text) were obtained from Gene Tex, Inc (North America). The antibody for rat of P-p38 (4511, CST) was obtained from Cell Signaling Technology (Shanghai, China). The antibody for rat of GAPDH (10494-1-AP, Proteintech) was purchased from Proteintech (Wuhan, China).

#### 2.2.2. Establishing the COPD Rat Model

A COPD rat model was performed in terms of previous studies [20]. SD rats were randomly classified to 4 groups: control group, COPD model group, I&N group, and doxofylline group. The COPD rat model was created via exposure to cigarette smoke (CSE) and *Klebsiella pneumoniae* infection. Specifically, the rats were exposed to CSE (3000 ± 500 ppm) for 40 minutes twice daily for 8 weeks and to *Klebsiella pneumoniae* (6 × 10^8^ CFU/ml, 0.1 ml) for 5 days once for 8 weeks. The procedures of this study were approved by the Experimental Animal Care and Ethics Committees of the First Affiliated Hospital of Henan University of Chinese Medicine, and the ethical review approval number is YFYDW2019031.

#### 2.2.3. Drug and Treatment

From week 9, the I&N group rats were given I&N at 2.12 mg/kg/d (the ratio of icariin to nobiletin was 12.5 : 1). The doxofylline is a newer generation xanthine, which is a kind of effective bronchodilator recommended by Global Initiative for Chronic Obstructive Lung Disease (GOLD) [2]. The doxofylline has beneficial effects with both bronchodilating and anti-inflammatory activities in COPD 1. So, we chose doxofylline as the control drug. The doxofylline group rats were given doxofylline at 36 mg/kg/d. The dosages of these drugs were calculated according the following formula (*D*: dose; *K*: body shape index, *K* = *A*/*W*^2/3^, *A*: surface area in m^2^, *W*: weight in kg):

(1) $$\begin{array}{c} D_{\text{rat}}=D_{\text{human}}\times \left(\frac{K_{\text{rat}}}{K_{\text{human}}}\right)\times {\left(\frac{W_{\text{rat}}}{W_{\text{human}}}\right)}^{2/3}. \end{array}$$

At week 17, 4 group rats were sacrificed after intraperitoneal injection of 2% pentobarbital sodium at 40 mg/kg.

#### 2.2.4. Lung Function Measurement and Lung Tissue Histopathology

Lung function was detected for all group rats every four weeks from 0 week to 16^th^ week via the tidal volume (TV), peak expiratory flow (PEF), and 50% tidal volume expiratory flow (EF50) by unrestrained pulmonary function testing plethysmographs (Buxco Inc., Wilmington, NC, USA).

The lung tissues were soaked in 4% paraformaldehyde solution. Next, the tissues were cut and embedded in paraffin and made slices. Then, the lung tissues slices were stained with hematoxylin and eosin and were observed by a light microscope (Olympus, Tokyo, Japan). The mean linear intercept (MLI) and mean alveolar numbers (MAN) were considered as the degree of alveolar damage. Under microscopy (×200), 6 visual fields were taken in each slice, and the alveolar number and the linear intercept in a fixed area of visual field were measured. MAN (/mm^2^) = Na/*A*. Na is the number of pulmonary alveoli in each visual field. *A* is the area of the visual field. Then, we made a cross (+) under the visual field and counted the number of alveolar septaon the cross. MLI (*μ*m) = *L*/Ns. Ns is the number of alveolar septa. *L* is total length of the cross.

#### 2.2.5. ELISA

The lung tissue was homogenized in PBS solution and centrifuged to collect the supernatant. The secretion of TNF-*α*, IL-1*β*, and IL-6 in a lung tissue homogenate was measured using ELISA kits, according to the manufacturer instructions. The dilution ratio of the lung tissue homogenate was determined according to the standard curve. Samples were incubated with antibodies in 96-well plates. The OD value was detected by a microplate reader (Thermo Fisher Scientific 1500, Vantaa, Helsinki, Finland), and the concentration was calculated according to the standard curve.

#### 2.2.6. Real-Time Polymerase Chain Reaction Assay

The mRNA levels of GAPDH (forward: ACAGCAACAGGGTGGTGGAC, reverse: TTTGAGGGTGCAGCGAACTT), TNF-*α* (forward: CGTCAGCCGATTTGCCATTT, reverse: TCCCTCAGGGGTGTCCTTAG), IL-1*β* (forward: CCTATGTCTTGCCCGTGGAG, reverse: CACACACTAGCAGGTCGTCA), and IL-6 (forward: TCCGGAGAGGAGACTTCACA, reverse: TTCTGACAGTGCATCATCGCT) in lung tissues were detected by qPCR.

#### 2.2.7. Western Blotting Assay

The lung tissues were lysed with RIPA buffer in ice to obtain protein samples. The concentrations of lung tissue protein samples were measured using BCA kits, and the lung tissue protein samples were adjusted to equal concentrations. The lung tissue protein samples with equal concentrations in each group were divided by SDS-PAGE electrophoresis and metastasized to PVDF membranes. 5% skim milk was used to block the PVDF. Next, membranes were incubated with their primary antibodies, including GAPDH (1 : 5000), P-p38 (1 : 1000), P13K (1 : 1000), and P-AKT (1 : 1000), and secondary antibodies (1 : 5000). The membranes were visualized using the Bio-Rad Imaging System (Pierce, USA).

#### 2.2.8. Statistical Analysis

The experimental data were analyzed by SPSS v21.0. A comparison among groups was performed by one-way analysis of variance with an appropriate post-hoc test. If the variances were homogeneous, the LSD method was performed. If the variances were inconsistent, Dunnett's T3 test was performed. The mean ± SD were used as the data present presentation. A *p* value of <0.05 was set for a statistically significant difference.

## 3. Results

### 3.1. Network Pharmacology

#### 3.1.1. Screening Targets of Components and COPD

**Figure 2:**
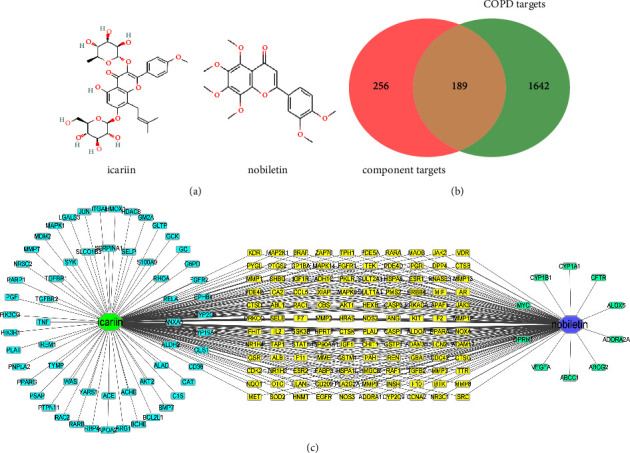
Network pharmacology analysis of I&N targets and COPD targets. (a) The 2D structures of I&N. (b) The Venn of I&N targets and COPD targets. (c) The component-target network.

From PubChem, the 2D structures of icariin and nobiletin were downloaded (Figure 2(a)). 445 genes were obtained as potential targets of icariin and nobiletin from SwissTargetPrediction database, STITCH database, and PharmMapper database. Then, 1,831 related genes of COPD were obtained from DisGeNET database, GeneCards database (score >15.0), and DrugBank database. Matching COPD targets with icariin and nobiletin targets, 189 genes (Figure 2(b)) were chosen as related genes of I&N against COPD for constructing the component-target (C-T) network (Figure 2(c)). The C-T network was built by Cytoscape software. According to the C-T network, 120 potential targets were common targets of icariin and nobiletin. 59 potential targets were unique targets of icariin and 10 potential targets of nobiletin.

#### 3.1.2. Protein-Protein Interaction (PPI) Network Analysis

**Figure 3:**
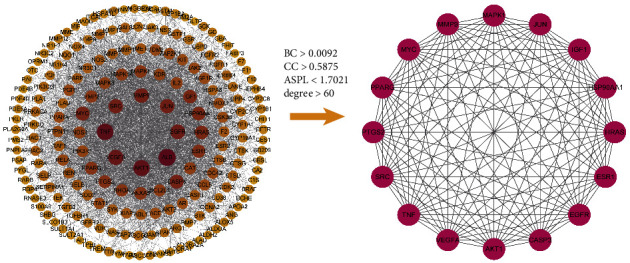
The PPI network of 189 targets and 16 key targets were screened by PPI network analysis.

All of 189 potential therapeutic targets were submitted to STRING database, and they were submitted to CytoScape3.8.2 for constructing and analyzing the PPI network (Figure 3). The PPI network consisted of 189 nodes and 2809 edges and the average degree was 23. Then, the targets with degree higher than double average degree were selected and 41 targets were screened for further analysis. Next, the mean value of BC, CC, ASPL, and degree of 41 targets were calculated using the Analyze Network tool of Cytoscape3.8.2. The targets with values of BC, CC, and degree higher than the mean value of BC, CC, and degree (BC > 0.0092, CC > 0.5875, degree > 60), and value of ASPL lower than mean value of ASPL (ASPL < 1.7021), were selected as key targets. Finally, 16 targets were screened out, including TNF, AKT1, VEGFA, EGFR, JUN, SRC, MMP9, CASP3, MYC, IGF1, HSP90AA1, HRAS, ESR1, PTGS2, PPARG, and MAPK1 (Figure 3).

#### 3.1.3. Enrichment Analysis of the GO and KEGG Pathways

**Figure 4:**
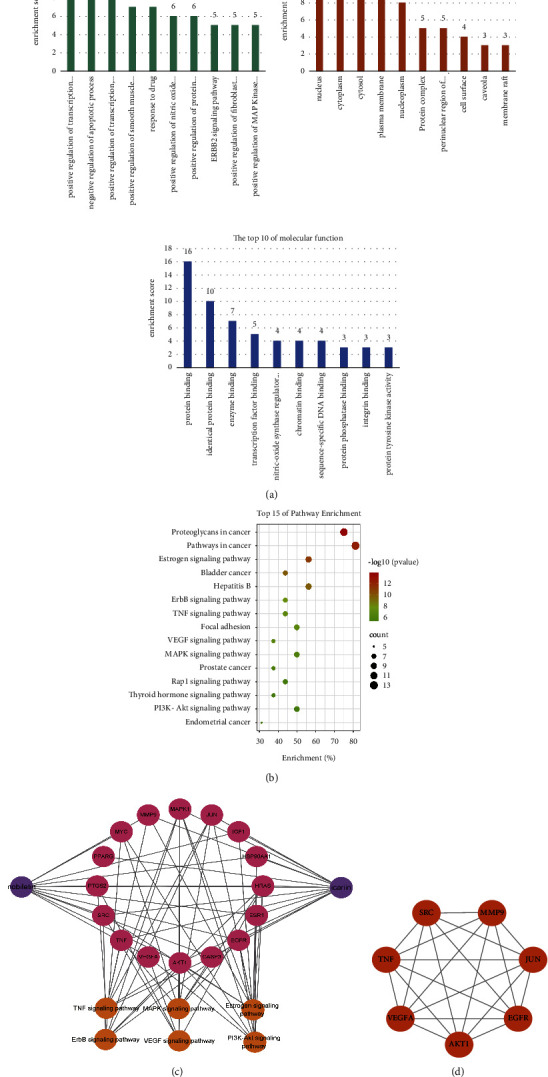
Network pharmacology analysis of common targets. (a) GO analysis of 16 key targets. (b) KEGG analysis of 16 key targets. (c) The component-target-pathway network. (d) 7 highest scoring targets according degree score.

The DAVID 6.8 database was used to perform GO and KEGG analyses on 16 important targets. Positive regulation of transcription from the RNA polymerase II promoter, negative regulation of the apoptotic process, and positive regulation of transcription, DNA-templated, were mostly enriched in BP enrichment analysis; nucleus, cytoplasm, and cytosol were mostly enriched in CC enrichment analysis; protein binding, identical protein binding, and enzyme binding were mostly enriched in MF analysis. (Figure 4(a)). The results of KEGG analysis indicated that the regulatory pathway included TNF, MAPK, and PI3K-Akt pathways (Figure 4(b)). These results suggested that I&N may exert inhibition effects of inflammation in COPD by regulating the TNF, MAPK, and PI3K-Akt pathways. The component-target-pathway network was built by Cytoscape software (Figure 4(c)).

#### 3.1.4. Molecular Docking

To clarify the potential interaction between two components and the key proteins, molecular docking was performed to reveal the possible binding mode between the 7 highest scoring proteins, including TNF, AKT1, VEGFA, EGFR, JUN, SRC, and MMP9 (Figure 4(d)), and two components. The binding energy was considered as an important factor for constituents screening (Table 1). Icariin was predicted to interact with AKT via 3 residues (ASP-119, GLN-59, and LEU-78), with EGFR via 6 residues (PRO-669, ASN-700, ARG-831, ARG-776, ILE-1018, and TYR-1016), with MMP9 via 6 residues (ARG-370, LEU-35, LYS-184, ASN-38, ASP-185, and THR-96), with JUN via 4 residues (DG-26, DG-27, DA-23, and DA-17), with SRC via 2 residues (GLU-270 and GLU-265), and with TNF via 4 residues (PHE-144, GLY-24, ASP-140, and PRO-139). In addition, nobiletin could bind to AKT by 3 residues (GLN-79, LEU-78, and GLN-59), to EGFR by 5 residues (VAL-769, ARG-776, ALA-767, LEU-777, and ILE-1018), to MMP9 by 4 residues (THR-426, GLY-428, PRO-430, and LEU-431), to SRC by 2 residues (TRP-260 and LYS-316), and to VEGFA by 2 residues (CYS-131, TYR-52).

**Figure 5:**
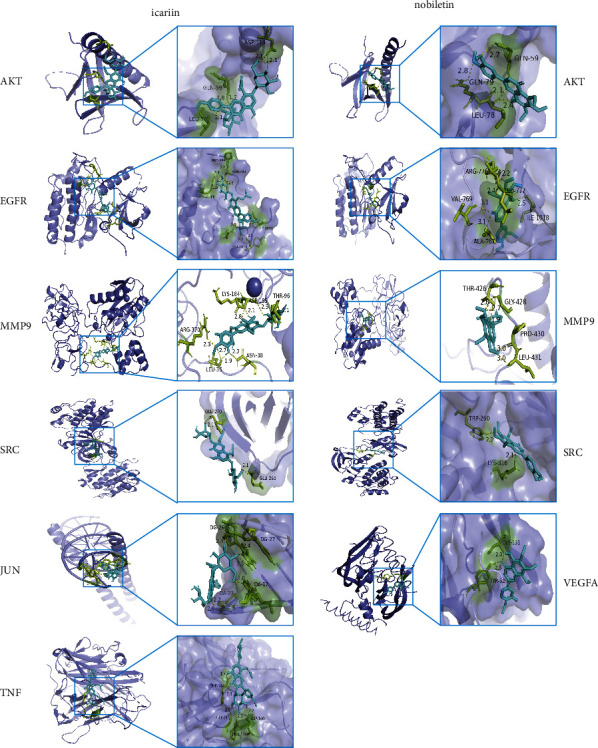
Molecular docking results: Icariin-AKT;icariin-EGFR;icariin-MMP9;icariin-JUN;icariin-SRC;icariin-TNF;nobiletin-AKT;nobiletin-EGFR;nobiletin-MMP9;nobiletin-SRC; and nobiletin-VEGFA.

According to Table 1 and Figure 5, icariin and nobiletin have strong binding interactions with TNF, AKT1, VEGFA, EGFR, JUN, SRC, and MMP9.

### 3.2. Experiment Validation

#### 3.2.1. Effects of I&N on the Lung Function in COPD Rats

**Figure 6:**
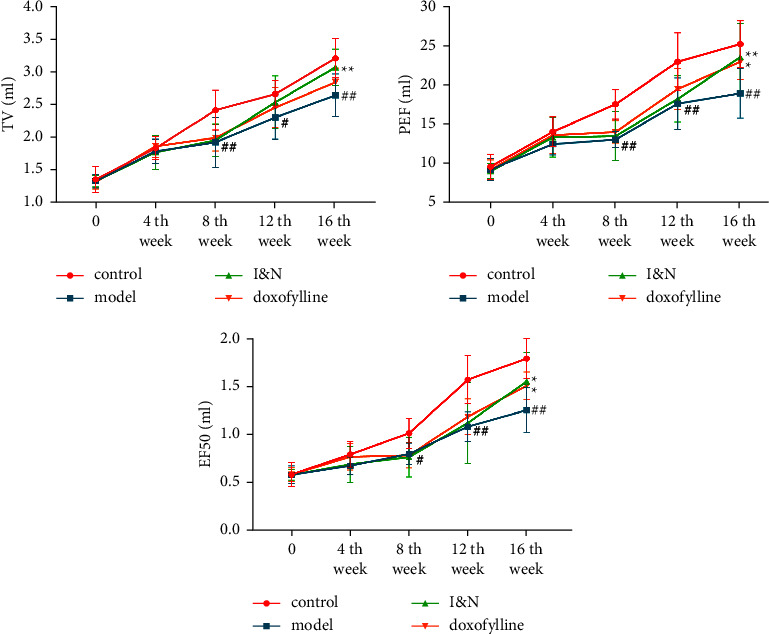
The TV, PEF, and EF50 in the lung function. The mean ± SD was used as the expression of values. ^##^*P* < 0.01, ^#^*P* < 0.05, compared to the control group. ^*∗∗*^*P* < 0.01, ^*∗*^*P* < 0.05, compared to the model group. *N* = 8.

To verify treatment of I&N of COPD, we established the COPD model through co-treatment with CSE and *Klebsiella pneumoniae* in rats. As described in Figure 6, compared with the control group, the TV, PEF, and EF50 in lung functions descended significantly in COPD rats (*P* < 0.05), and I&N and doxofylline increased the TV, PEF, and EF50 in rats (*P* < 0.05).

#### 3.2.2. Effects of I&N on Lung Tissue Histopathology in COPD Rats

**Figure 7:**
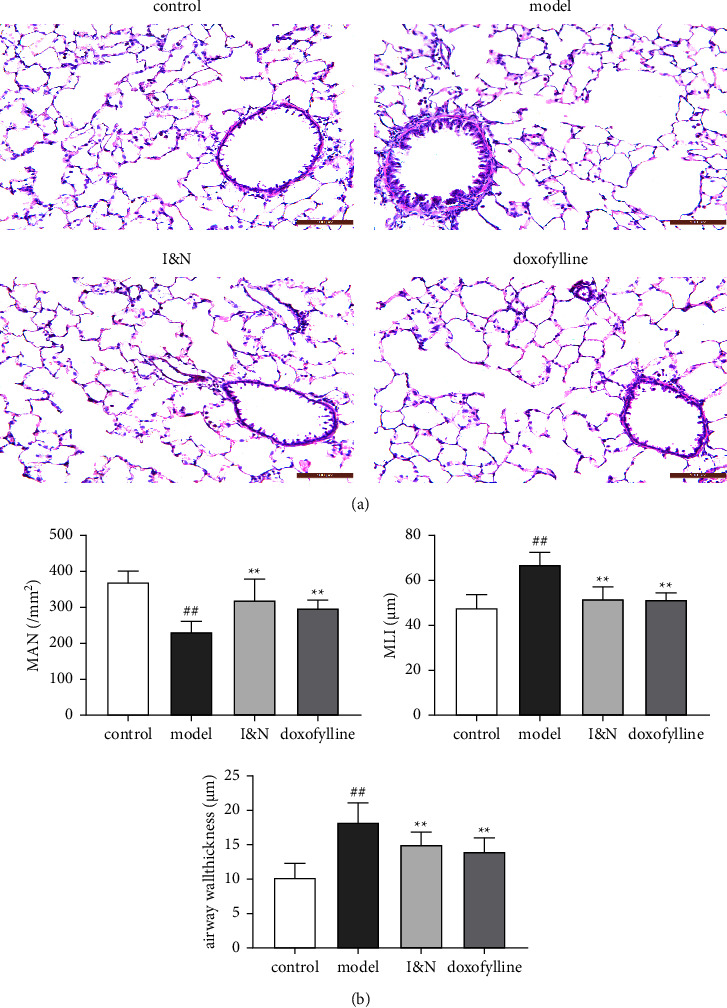
The effects of I&N in COPD rats. (a) Hematoxylin and eosin staining (HE), x200 magnification times. (b) Lung tissue histopathology quantitative analysis. The mean ± SD was used as the expression of values. ^##^*P* < 0.01, ^#^*P* < 0.05, compared to the control group. ^*∗∗*^*P* < 0.01, ^*∗*^*P* < 0.05, compared to the model group. *N* = 8.

Lung tissue histopathology analysis indicates that I&N reduced alveolar damage and airway wall thickness (Figure 7(a)). Quantitative analysis of lung tissue histopathology showed that (Figure 7(b)), compared to the control group, MAN was decreased and MLI was increased in COPD rats (*P* < 0.05); I&N and doxofylline increased MAN and decreased MLI (*P* < 0.05); and I&N effectively relieved the thickened airway wall in COPD rats (*P* < 0.05).

#### 3.2.3. Effect of I&N on the Inflammatory Response and Inflammation-Related Pathway

**Figure 8:**
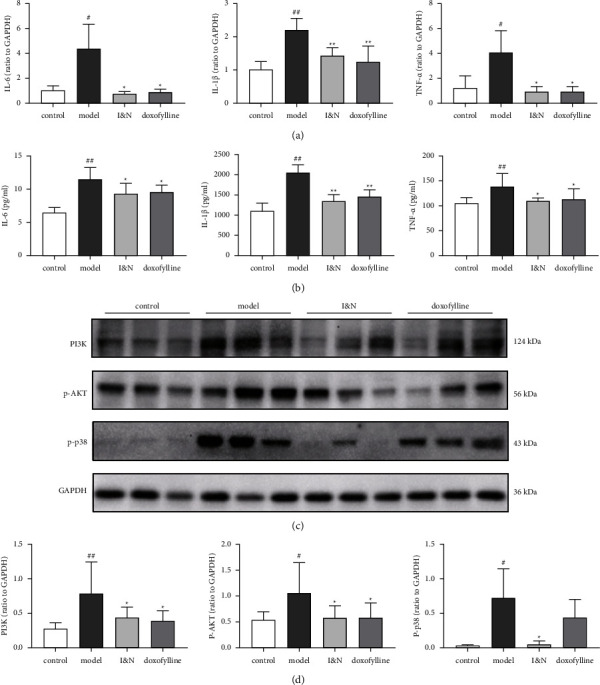
The anti-inflammation effects of I&N in COPD rats. (a) The mRNA levels of IL-6, IL-1*β*, and TNF-*α* in lung tissues. (b) The expression of IL-6, IL-1*β*, and TNF-*α* in lung tissues. (c) The western blot bands of PI3K, P-AKT, and P-p38. (d) Quantitative analysis of western blot bands. *N* = 6.

In the COPD rats, the mRNA levels and protein secretion of inflammatory factor in lung tissues were significantly increased, including IL-6, IL-1*β*, and TNF-*α*, and these were decreased with treatment of I&N (*P* < 0.05) (Figures 8(a) and 8(b)). As in Figures 8(c) and 8(d), the expression of PI3K, P-AKT, and P-p38 of lung tissues were significantly increased in the model group, and I&N decreased the expression of PI3K and phosphorylation of P-AKT and P-p38 of lung tissues in COPD rats (*P* < 0.05). These results suggested that I&N inhibit inflammatory responses in COPD rats via regulating the PI3K-AKT and MAPK pathways.

## 4. Discussion

It has been verified that TCM has positive therapeutic effects on COPD. BYF, a TCM therapeutic strategy for COPD, has demonstrated that it can inhibit secretion of inflammatory cytokine, recover protease-antiprotease imbalance, and reduce collagen deposition [22]. Due to the complicacy of TCM ingredients, it is difficult to explore potential therapeutic mechanisms of BYF. Therefore, five critical active ingredients of BYF were screened out and integrated into effective-component compatibility of Bufei Yishen formula (ECC-BYF), including icariin, nobiletin, astragaloside IV, 20-S-ginsenoside Rh1, and paeonol. It has been verified the treatment effects of ECC-BYF for COPD on improving the pulmonary function and reducing pathological damage and the inflammatory cytokine levels in lung tissues in COPD rats [23]. Icariin and nobiletin are two main active ingredients of ECC-BYF. In a previous study, we found the effects of I&N on improving the lung function, reducing pathological damage, and inhibiting inflammatory response in COPD rats. However, the therapeutic mechanisms of I&N for COPD remain unclear. In this study, we devote to reveal the treatments and mechanisms of I&N against COPD. Therefore, we integrated network pharmacology and experiment verification to systematically evaluate the potential pharmacological mechanisms of I&N for COPD.

First, we applied network pharmacology to screen the possible targets of I&N against COPD. 189 targets of I&N in COPD were obtained from 6 databases, and those with BC > 0.0092, CC > 0.5875, degree > 60, and ASPL < 1.7021 were considered as key targets. 16 key targets were screened out from the 189 targets via PPI network analysis, including TNF, AKT, and MAPK1. These key targets were significantly related to inflammation. Furthermore, the 16 key targets were mostly enriched inflammation-related pathway according GO analysis and KEGG analysis, such as TNF, PI3K-AKT, and MAPK signaling pathways. The result suggested I&N may inhibit the inflammatory response in COPD via these proteins and pathways. Then, molecular docking of I&N and these proteins was performed to verify the possibility of interaction, and these proteins, including AKT, TNF, EGFR, and MMP9, had strong binding energy with I&N.

Inflammation is a key pathological reaction for the development of COPD [24]. The main inflammatory cells in COPD involve neutrophils, macrophages, and lymphocytes in the lung tissue and airway [25]. The inflammatory mediators and destructive enzymes from inflammatory cells are related to the structural damage of the airway and lung tissue in COPD [26]. For instance, neutrophils in COPD patients and COPD model rats are recruited to the lung and airway and secrete various serine proteases, including myeloperoxidase (MPO), matrix metalloproteinase (MMP), and neutrophil elastase (NE), all of which are related to destruction of the alveolar airway and cause emphysema [27]. PI3K, a kind of lipid kinases, induced the phosphorylation of AKT to regulate cell survival, growth, multiplication, and death in response to extracellular signals. Based on previous studies, the inflammatory efficacy of the PI3K-AKT signaling pathway in COPD. The concentrations of TNF-*α* and IL-6 in both the bronchoalveolar lavage fluid (BALF) and serum are decreased via restraining the activation of PI3K-AKT signaling in COPD model rats [28]. Macrolide reduces lung and systemic inflammation of COPD patients by regulating the PI3K-AKTN pathway [29]. The family of MAPKs, including p38, ERK, and JNK, is considered as a significant role in the inflammatory process [30]. The MAPK signaling pathway regulates COPD-related characteristics such as chronic inflammation and cytokine expression. The levels of phosphorylation of ERK, p38, and JNK in RAW 264.7 cells stimulated by CSE are much higher, indicating that MAPK signaling was activated in macrophages. Treatment with a MAPK signaling inhibitor also successfully inhibited the TNF-*α*, IL-1*β*, and HO-1 overexpression following CSE [31]. Moreover, PI3K-AKT and MAPK signaling pathways are considered as the major pathways, which observably upregulate the MUC5AC expression with the elevated phosphorylation level [32]. MUC5AC, a major secreted mucin which is closely connected with the viscoelasticity of sputum, endangers mucociliary functions and decreases mucus clearance because of secretion excessive, and leads to aggravated lung infection [33]. According to previous studies, the secretion of MUC5AC was downregulated via the inhibition of PI3K-AKT signaling pathway phosphorylation [34].

It has been reported that icariin inhibits CSE-induced inflammation, ROS production, and airway remodeling via mitigating glucocorticoids resistance in CSE-exposed BEAS-2B cells [35]. In addition, nobiletin exhibited protective effects in decreasing the production of TNF-*α*, IL-6 via restraining activation of NF-*κ*B signaling in the LPS-induced acute lung injury mice model and LPS-stimulated A549 cells [36]. We had validated the anti-inflammatory effect of I&N against COPD in in vivo experiment. The mRNA and protein expression levels of IL-6, IL-1*β*, and TNF-*α* in lung tissues of COPD model rats were significantly increased and were decreased by I&N and doxofylline. On the other hand, the decline of lung function and emphysema is a common symptom during the development of COPD [37]. In in vivo experiments, the lung function and alveolar damage were significantly improved by treatment of I&N and doxofylline compared to the model group. Furthermore, the expression levels of PI3K and phosphorylation levels of P-AKT and P-p38 in lung tissues were significantly decreased after the treatment of I&N and doxofylline compared to the model group. These results confirm the inhibition inflammatory response effects of I&N in by decreasing the expression levels of inflammatory cytokines. Moreover, doxofylline can improve the lung function and the expression of inflammatory factors in COPD rats. The therapeutic effects of I&N were consistent with those of doxofylline in improving symptoms and inhibiting inflammation. Moreover, the potential mechanism may be related to suppress the phosphorylation of the PI3K-AKT and MAPK pathway in COPD.

## 5. Conclusion

In our research, the therapeutic efficacy and mechanisms of I&N for COPD are verified via the method integrating network pharmacology and experiment validation. 16 key targets of I&N against COPD were screened, including TNF, AKT1, and MAPK1. According KEGG pathway analysis, the activation of the MPAK and PI3K-AKT pathways was a significant mechanism of I&N against COPD. In in vivo experiments, the lung function, pathological damage of lung tissues, and secretion of IL-6, IL-1*β*, and TNF-*α* were improved by treatment of I&N in COPD rats. Furthermore, the levels of PI3K, P-AKT, and P-p38 were reduced by I&N. In conclusion, I&N have significant anti-inflammation effects for COPD via the restraining activation of PI3K-AKT and MPAK pathways. However, the complex mechanisms of I&N for treatment of COPD require further exploring.

## Figures and Tables

**Table 1:** Binding energy between components and 7 highest scoring targets (kcal/mol).

Components	CID	Targets	PDB ID	Binding energy
Icariin	5318997	AKT	2UZR	−4.38
Icariin	5318997	EGFR	5Y9T	−5.84
Icariin	5318997	MMP9	1L6J	−5.32
Icariin	5318997	JUN	5T01	−3.25
Icariin	5318997	SRC	2BDF	−2.95
Icariin	5318997	TNF	7KP9	−4.28
Nobiletin	72344	AKT	2UZR	−5.36
Nobiletin	72344	EGFR	5Y9T	−5.85
Nobiletin	72344	MMP9	1L6J	−6.2
Nobiletin	72344	SRC	2BDF	−6.18
Nobiletin	72344	VEGFA	7LL8	−5.84

## Data Availability

The data used to support the findings of this study can be obtained from the corresponding author according to the rules.

## Supplementary Materials

Supplementary Materials The authors provided supplementary information on 189 common targets.
