# Supplementary material for: Network Pharmacology and Experimental Validation to Reveal Effects and Mechanisms of Icariin Combined with Nobiletin against Chronic Obstructive Pulmonary Diseases
Source: Evid Based Complement Alternat Med. 2022 Nov 3;2022:4838650. doi: 10.1155/2022/4838650 (PMC9649313; doi:10.1155/2022/4838650)
Supplement: Supplementary Materials — The authors provided supplementary information on 189 common targets. [file 4838650.f1.zip › I&N targets.pdf]

number	targets	ingredient
1	ABL1	common
2	ABO	common
3	ADAM17	common
4	ADAM33	common
5	ADH1C	common
6	ADH5	common
7	ADK	common
8	ADORA1	common
9	AHCY	common
10	AK1	common
11	AKR1B1	common
12	AKR1C1	common
13	AKR1C3	common
14	AKT1	common
15	ALB	common
16	ALDOA	common
17	AMD1	common
18	AMY2A	common
19	ANG	common
20	APAF1	common
21	APCS	common
22	APRT	common
23	AR	common
24	ARL5A	common
25	ARL5B	common
26	ATIC	common
27	B3GAT1	common
28	BACE1	common
29	BCAT2	common
30	BHMT	common
31	BIRC7	common
32	BRAF	common
33	BST1	common
34	BTK	common
35	CA12	common
36	CA2	common
37	CASP1	common
38	CASP3	common
39	CBR1	common
40	CBS	common
41	CCL5	common
42	CCNA2	common
43	CCNT1	common
44	CD1A	common
45	CD209	common
46	CDA	common
47	CDC42	common
48	CDK2	common
49	CDK6	common
50	CDK7	common
51	CFB	common
52	CHEK1	common
53	CHIT1	common

54	CLEC4M	common
55	CLK1	common
56	CMA1	common
57	CTSB	common
58	CTSD	common
59	CTSF	common
60	CTSG	common
61	CTSK	common
62	CTSS	common
63	CYP2C9	common
64	DAPK1	common
65	DCK	common
66	DCPS	common
67	DCXR	common
68	DDX39B	common
69	DHFR	common
70	DHODH	common
71	DOT1L	common
72	DPEP1	common
73	DPP4	common
74	DTYMK	common
75	DUT	common
76	EEA1	common
77	EGFR	common
78	EIF4E	common
79	ELANE	common
80	ERBB4	common
81	ERI1	common
82	ESR1	common
83	ESR2	common
84	ESRRG	common
85	F10	common
86	F11	common
87	F2	common
88	F7	common
89	FABP3	common
90	FABP6	common
91	FDPS	common
92	FECH	common
93	FGFR1	common
94	FGG	common
95	FHIT	common
96	FKBP1A	common
97	FKBP1B	common
98	FKBP3	common
99	FNTA	common
100	GART	common
101	GBA	common
102	GCDH	common
103	GMPR	common
104	GMPR2	common
105	GP1BA	common
106	GPI	common
107	GSK3B	common

108	GSR	common
109	GSTA1	common
110	GSTA3	common
111	GSTM1	common
112	GSTM2	common
113	GSTO1	common
114	GSTP1	common
115	GSTT2B	common
116	GSTZ1	common
117	HADH	common
118	HAGH	common
119	HCK	common
120	HEXB	common
121	HINT1	common
122	HK1	common
123	HMGCR	common
124	HNMT	common
125	HPGDS	common
126	HPRT1	common
127	HRAS	common
128	HSD11B1	common
129	HSD17B1	common
130	HSP90AA1	common
131	HSPA1L	common
132	HSPA8	common
133	IGF1	common
134	IGF1R	common
135	IL2	common
136	IMPDH1	common
137	IMPDH2	common
138	INSR	common
139	ISG20	common
140	ITK	common
141	ITPKA	common
142	JAK2	common
143	JAK3	common
144	KAT2B	common
145	KDR	common
146	KIF11	common
147	KIT	common
148	LCK	common
149	LCN2	common
150	LGALS2	common
151	LGALS7	common
152	LSS	common
153	LTA4H	common
154	LYZ	common
155	MAN1B1	common
156	MAOB	common
157	MAP2K1	common
158	MAPK10	common
159	MAPK14	common
160	MAPK8	common
161	MAPKAPK2	common

162	ME2	common
163	MET	common
164	METAP2	common
165	MIF	common
166	MME	common
167	MMP1	common
168	MMP12	common
169	MMP13	common
170	MMP2	common
171	MMP3	common
172	MMP8	common
173	MMP9	common
174	MTAP	common
175	NDST1	common
176	NMNAT1	common
177	NMNAT3	common
178	NOS2	common
179	NOS3	common
180	NOX4	common
181	NQO1	common
182	NR1H2	common
183	NR1H3	common
184	NR1H4	common
185	NR1I2	common
186	NR3C1	common
187	NT5M	common
188	OAT	common
189	OTC	common
190	PAH	common
191	PAPSS1	common
192	PCK1	common
193	PDE4B	common
194	PDE4D	common
195	PDE5A	common
196	PDK2	common
197	PDPK1	common
198	PFKFB1	common
199	PGR	common
200	PIM1	common
201	PITPNA	common
202	PKLR	common
203	PLA2G2A	common
204	PLAU	common
205	PLEKHA4	common
206	PMS2	common
207	PNMT	common
208	PNP	common
209	PPARA	common
210	PPARD	common
211	PPP1CC	common
212	PRKACA	common
213	PRKCQ	common
214	PTGS2	common
215	PTPN1	common

216	PYGL	common
217	RAB5A	common
218	RAB9A	common
219	RAC1	common
220	RAF1	common
221	RAN	common
222	RAP2A	common
223	RARA	common
224	REN	common
225	RFK	common
226	RHEB	common
227	RNASE2	common
228	RNASE3	common
229	RNASE4	common
230	RND3	common
231	RXRA	common
232	RXRB	common
233	SDS	common
234	SELE	common
235	SHBG	common
236	SHMT1	common
237	SIRT5	common
238	SOD2	common
239	SORD	common
240	SPR	common
241	SRC	common
242	SSE1	common
243	STAT1	common
244	SULT1A1	common
245	SULT1E1	common
246	SULT2A1	common
247	SULT2B1	common
248	TAP1	common
249	TEK	common
250	TGFB2	common
251	TGM3	common
252	THRB	common
253	TPH1	common
254	TPI1	common
255	TPSB2	common
256	TRDMT1	common
257	TTR	common
258	TYMS	common
259	UAP1	common
260	UCK2	common
261	UMPS	common
262	VDR	common
263	WARS1	common
264	XIAP	common
265	ZAP70	common
I-1	ACADM	icariin
I-2	ACAT1	icariin
I-3	ACE	icariin
I-4	ACHE	icariin

I-5	ACP3	icariin
I-6	ADH1B	icariin
I-7	ADRA2A	icariin
I-8	ADRA2C	icariin
I-9	AGXT	icariin
I-10	AKR1C2	icariin
I-11	AKT2	icariin
I-12	ALAD	icariin
I-13	ALDH2	icariin
I-14	AMY1A	icariin
I-15	ANXA5	icariin
I-16	APOA2	icariin
I-17	ARF1	icariin
I-18	ARF4	icariin
I-19	ARG1	icariin
I-20	ARG2	icariin
I-21	ARHGAP1	icariin
I-22	ATOX1	icariin
I-23	AZGP1	icariin
I-24	BAG1	icariin
I-25	BCHE	icariin
I-26	BCL2L1	icariin
I-27	BLVRB	icariin
I-28	BMP7	icariin
I-29	C1R	icariin
I-30	C1S	icariin
I-31	CA1	icariin
I-32	CA4	icariin
I-33	CA7	icariin
I-34	CAMKK2	icariin
I-35	CANT1	icariin
I-36	CASP7	icariin
I-37	CAT	icariin
I-38	CD38	icariin
I-39	CDK5R1	icariin
I-40	CES1	icariin
I-41	CFD	icariin
I-42	CLC	icariin
I-43	CPB1	icariin
I-44	CRABP2	icariin
I-45	CRAT	icariin
I-46	CRYZ	icariin
I-47	CSK	icariin
I-48	CSNK1G2	icariin
I-49	CSNK2A1	icariin
I-50	CTNNA1	icariin
I-51	CTSL	icariin
I-52	CTSV	icariin
I-53	CYP19A1	icariin
I-54	CYP2C8	icariin
I-55	DDX6	icariin
I-56	EPHA2	icariin
I-57	EPHB4	icariin
I-58	EPHX2	icariin

I-59	ESRRA	icariin
I-60	FABP4	icariin
I-61	FABP5	icariin
I-62	FABP7	icariin
I-63	FAP	icariin
I-64	FCAR	icariin
I-65	FGFR2	icariin
I-66	FOLH1	icariin
I-67	G6PD	icariin
I-68	GALE	icariin
I-69	GALK1	icariin
I-70	GC	icariin
I-71	GCK	icariin
I-72	GLO1	icariin
I-73	GLTP	icariin
I-74	GM2A	icariin
I-75	GNPDA1	icariin
I-76	GRB2	icariin
I-77	HDAC8	icariin
I-78	HMOX1	icariin
I-79	HNF4G	icariin
I-80	HSD17B11	icariin
I-81	HSP90AB1	icariin
I-82	ICAM2	icariin
I-83	IGLV2-8	icariin
I-84	IMPA1	icariin
I-85	ITGAL	icariin
I-86	IVD	icariin
I-87	JUN	icariin
I-88	KYAT1	icariin
I-89	LDHB	icariin
I-90	LGALS3	icariin
I-91	MAOA	icariin
I-92	MAPK1	icariin
I-93	MAPK12	icariin
I-94	MDM2	icariin
I-95	MMP16	icariin
I-96	MMP7	icariin
I-97	MTHFD1	icariin
I-98	NCS1	icariin
I-99	NME2	icariin
I-100	NMUR2	icariin
I-101	NQO2	icariin
I-102	NR1I3	icariin
I-103	NR3C2	icariin
I-104	NUDT9	icariin
I-105	PADI4	icariin
I-106	PAK6	icariin
I-107	PAK7	icariin
I-108	PARP1	icariin
I-109	PCTP	icariin
I-110	PDE11A	icariin
I-111	PDE3B	icariin
I-112	PGF	icariin

I-113	PIK3CG	icariin
I-114	PIK3R1	icariin
I-115	PLA2G10	icariin
I-116	PLAT	icariin
I-117	PLK1	icariin
I-118	PNPLA2	icariin
I-119	PNPO	icariin
I-120	PPARG	icariin
I-121	PPCDC	icariin
I-122	PPIA	icariin
I-123	PROCR	icariin
I-124	PSAP	icariin
I-125	PTK2	icariin
I-126	PTPN11	icariin
I-127	QPCT	icariin
I-128	RAB11A	icariin
I-129	RAC2	icariin
I-130	RARB	icariin
I-131	RARG	icariin
I-132	RBP4	icariin
I-133	REG1A	icariin
I-134	RELA	icariin
I-135	RHOA	icariin
I-136	RORA	icariin
I-137	RPS6KA3	icariin
I-138	S100A9	icariin
I-139	SCARB1	icariin
I-140	SEC14L2	icariin
I-141	SELP	icariin
I-142	SERPINA1	icariin
I-143	SETD7	icariin
I-144	SETD8	icariin
I-145	SLCO1B3	icariin
I-146	SLCO2B1	icariin
I-147	SQLE	icariin
I-148	SRM	icariin
I-149	ST14	icariin
I-150	STK6	icariin
I-151	STS	icariin
I-152	SYK	icariin
I-153	TGFBR1	icariin
I-154	TGFBR2	icariin
I-155	THRA	icariin
I-156	TK1	icariin
I-157	TNF	icariin
I-158	TNK2	icariin
I-159	TNNC1	icariin
I-160	TRAPPC3	icariin
I-161	TREM1	icariin
I-162	TTPA	icariin
I-163	TYMP	icariin
I-164	WAS	icariin
I-165	YARS1	icariin
N-1	ABCC1	nobiletin

N-2	ABCG2	nobiletin
N-3	ADORA2A	nobiletin
N-4	ADORA3	nobiletin
N-5	ALOX5	nobiletin
N-6	AURKA	nobiletin
N-7	CFTR	nobiletin
N-8	CYP1A1	nobiletin
N-9	CYP1B1	nobiletin
N-10	DGAT1	nobiletin
N-11	MYC	nobiletin
N-12	OPRD1	nobiletin
N-13	OPRM1	nobiletin
N-14	TMPRSS11D	nobiletin
N-15	VEGFA	nobiletin
