# Supplementary material for: Network Pharmacology and Experimental Validation to Reveal Effects and Mechanisms of Icariin Combined with Nobiletin against Chronic Obstructive Pulmonary Diseases
Source: Evid Based Complement Alternat Med. 2022 Nov 3;2022:4838650. doi: 10.1155/2022/4838650 (PMC9649313; doi:10.1155/2022/4838650)
Supplement: Supplementary Materials — The authors provided supplementary information on 189 common targets. [file 4838650.f1.zip › I&N-COPD-targets.pdf]

189 targets
ABCC1
ABCG2
ABL1
ACE
ACHE
ADAM17
ADAM33
ADH1C
ADORA1
ADORA2A
AKT1
AKT2
ALAD
ALB
ALDH2
ALDOA
ALOX5
ANG
ANXA5
APAF1
APOA2
AR
ARG1
BCHE
BCL2L1
BMP7
BRAF
BTK
C1S
CA2
CASP1
CASP3
CAT
CBS
CCL5
CCNA2
CD209
CD38
CDC42
CDK2
CES1
CFTR
CHIT1
CTSB
CTSD
CTSG
CTSK
CYP19A1
CYP1A1
CYP1B1
CYP2C8
CYP2C9
DPP4

EGFR
ELANE
EPHB4
ERBB4
ESR1
ESR2
F10
F11
F2
F7
FABP3
FGFR1
FGFR2
FHIT
G6PD
GBA
GC
GCK
GLTP
GM2A
GP1BA
GSK3B
GSR
GSTM1
GSTP1
HDAC8
HEXB
HMGCR
HMOX1
HNMT
HPRT1
HRAS
HSP90AA1
HSPA1L
HSPA8
IGF1
IGF1R
IL2
INSR
ITGAL
JAK2
JAK3
JUN
KDR
KIT
LCN2
LGALS3
MAOB
MAP2K1
MAPK1
MAPK14
MAPK8
MDM2
MET

MIF
MME
MMP1
MMP12
MMP13
MMP2
MMP3
MMP7
MMP8
MMP9
MYC
NOS2
NOS3
NOX4
NQO1
NR1H3
NR1H4
NR3C1
NR3C2
OPRM1
OTC
PAH
PARP1
PDE4B
PDE4D
PDE5A
PGF
PGR
PIK3CG
PIK3R1
PKLR
PLA2G2A
PLAT
PLAU
PMS2
PNPLA2
PPARA
PPARG
PRKACA
PRKCQ
PSAP
PTGS2
PTPN11
PYGL
RAC1
RAC2
RAF1
RARA
RARB
RBP4
RELA
REN
RHOA
RNASE3

S100A9
SELE
SELP
SERPINA1
SHBG
SLCO1B3
SOD2
SRC
STAT1
SULT1A1
SULT2A1
SYK
TAP1
TEK
TGFB2
TGFBR1
TGFBR2
TNF
TPH1
TREM1
TTR
TYMP
VDR
VEGFA
WAS
XIAP
YARS1
ZAP70
