# Supplementary material for: Network Pharmacology and Experimental Validation to Reveal Effects and Mechanisms of Icariin Combined with Nobiletin against Chronic Obstructive Pulmonary Diseases
Source: Evid Based Complement Alternat Med. 2022 Nov 3;2022:4838650. doi: 10.1155/2022/4838650 (PMC9649313; doi:10.1155/2022/4838650)
Supplement: Supplementary Materials — The authors provided supplementary information on 189 common targets. [file 4838650.f1.zip › related genes of COPD.pdf]

number	elated genes
1	BMPR2
2	SERPINA1
3	TNF
4	TERT
5	IL6
6	MMP1
7	TP53
8	TGFB1
9	CYBB
10	SFTPC
11	CFTR
12	IL10
13	HLA-DRB1
14	ACE
15	PKD1
16	SFTPB
17	ABCA3
18	CYBA
19	RTEL1
20	NCF2
21	NOD2
22	IL1B
23	APOE
24	GBA
25	CRP
26	ENG
27	CTLA4
28	TEL1-TNFRSF6B
29	IFNG
30	PKD2
31	PKHD1
32	LMNA
33	MIR17
34	NCF1
35	CXCL8
36	ALB
37	RET
38	MUC5B
39	JAK2
40	GAA
41	EIF2AK4
42	ACVRL1
43	SFTPA1
44	KRAS
45	TLR4
46	NOS3
47	HLA-B
48	NCF4
49	HFE
50	VWF
51	GLA
52	NF1
53	STAT3

54	MPO
55	SMPD1
56	MIR21
57	SFTPA2
58	NPC1
59	CAV1
60	CCL2
61	IL13
62	VEGFA
63	PSEN1
64	MMP9
65	F2
66	HMOX1
67	FLNA
68	FAM13A
69	SNCA
70	ATM
71	PTPN11
72	EDN1
73	EGFR
74	ELN
75	STAT1
76	KIT
77	KCNK3
78	ABL1
79	APP
80	NLRP3
81	BRAF
82	DSP
83	PTEN
84	EDNRB
85	MPZ
86	REN
87	HLA-DQB1
88	SFTPD
89	PARN
90	IL4
91	TERC
92	SMAD4
93	FBN1
94	RYR1
95	NOTCH1
96	AKT1
97	PRKN
98	MFN2
99	BRCA2
100	MTHFR
101	TLR2
102	PTPN22
103	PMS2
104	POLG
105	ADRB2
106	LOC106627981
107	NPPB

108	F5
109	MUC1
110	LRRK2
111	CTNNB1
112	IL1RN
113	TRPV4
114	MAPT
115	GATA4
116	CCR6
117	NKX2-5
118	LOC110806263
119	FOXF1
120	ICAM1
121	IL17A
122	PDGFRB
123	SOD1
124	ELANE
125	MIR126
126	FAS
127	SERPINA3
128	HLA-DPB1
129	AGT
130	MIR34A
131	CCL11
132	INS
133	ATP7B
134	SETBP1
135	JAG1
136	SQSTM1
137	TSC2
138	BCR
139	FCGR2A
140	EDNRA
141	NAGLU
142	CCL5
143	IL2
144	SH3TC2
145	AGTR1
146	HBB
147	CCND1
148	IL2RA
149	SLC11A1
150	PSAP
151	MIR20A
152	TBX4
153	SLC17A5
154	MIR223
155	GDNF
156	PRTN3
157	PIK3CA
158	VCP
159	PMP22
160	TTR
161	ERBB2

162	SERPINE1
163	NEFL
164	IL18
165	PPARG
166	EDN3
167	GATA3
168	GJB1
169	GATA6
170	GDAP1
171	CSF2RA
172	IGHMBP2
173	LOC106029312
174	PDGFRA
175	MYH7
176	NCF4-AS1
177	COL1A1
178	APOA1
179	ABCA1
180	MIR15A
181	MIR145
182	IL5
183	ABCA4
184	SMAD9
185	CCR5
186	HRAS
187	LEP
188	SOX10
189	TNFRSF1A
190	UMOD
191	MIR29A
192	IL1A
193	MIF
194	CHAT
195	MIR155
196	TET2
197	FGFR3
198	COL4A5
199	WT1
200	ERBB3
201	HSPB1
202	SMAD3
203	MEFV
204	DYNC1H1
205	HNF1B
206	TSC1
207	SOS1
208	ACTG2
209	FGFR1
210	MAPK1
211	CP
212	ACTA2
213	NKX2-1
214	FN1
215	BDNF

216	CSF2
217	CD4
218	ADA
219	AGL
220	CYBC1
221	PL36A-HNRNPH2
222	COL4A4
223	MT-ND1
224	CFH
225	FIG4
226	TBX5
227	GPT
228	PARK7
229	MIR146A
230	APC
231	B2M
232	NRAS
233	TIMP1
234	EPO
235	HEXA
236	GUCY2C
237	IGF1
238	GARS1
239	RUNX1
240	CCL3
241	MBL2
242	NPC2
243	CCN2
244	MMP2
245	MYH11
246	TNFRSF11B
247	CEP290
248	IFNA1
249	F8
250	COPDA1
251	PTGS2
252	FOXP3
253	MMP3
254	CASR
255	HTT
256	ADIPOQ
257	MIR143
258	VDR
259	FGF10
260	FASLG
261	COMT
262	THBD
263	ACTC1
264	MMP12
265	ALMS1
266	ABCB4
267	FGFR2
268	TGFBR1
269	PON1

270	GSTM1
271	HLA-A
272	CLCN5
273	NOS2
274	HIF1A
275	ABCB1
276	NPPA
277	BRCA1
278	EGF
279	ALOX5
280	HLA-DQA1
281	CXCL10
282	TGFBR2
283	SERPINC1
284	LAMP2
285	AR
286	MME
287	GFAP
288	TTN
289	INPP5E
290	MIR106B
291	CSF3
292	NFKB1
293	PINK1
294	CD36
295	CASP3
296	TGFB2
297	CDKN2A
298	INF2
299	CD40LG
300	TBX1
301	ATP7A
302	ITGAM
303	CXCR4
304	ASXL1
305	SCN5A
306	GSTP1
307	AARS1
308	MT-CO1
309	LTA
310	PRKAG2
311	CASP8
312	CDH1
313	STN1
314	TNNI3
315	MKS1
316	SCGB1A1
317	SPINK1
318	RNASE3
319	GJA1
320	COL2A1
321	APOB
322	TNFRSF11A
323	MT-ATP6

324	SPP1
325	PYGM
326	MPL
327	CXCR3
328	F3
329	COL4A1
330	MTOR
331	IL23R
332	COL4A3
333	MYH6
334	MT-TL1
335	TNNT2
336	ACTA1
337	VHL
338	IDUA
339	CAT
340	IL10RA
341	CLEC7A
342	RAF1
343	IRF5
344	MUC5AC
345	MYC
346	GANAB
347	PLAU
348	DNM2
349	COL1A2
350	MIR204
351	VCAM1
352	DYNC2H1
353	CFTR-AS1
354	TYMP
355	LRRC56
356	CPT2
357	GDF1
358	FGF2
359	SLC6A4
360	SLC34A1
361	LTBP4
362	BMP6
363	DNAH5
364	APOL1
365	CD79A
366	TMEM67
367	IL17F
368	MGP
369	LIPA
370	PLAT
371	SDHB
372	PLP1
373	F9
374	KIF1B
375	ESR1
376	MAP2K1
377	GALC

378	CALCA
379	EGR2
380	CC2D2A
381	MECP2
382	FGF23
383	THPO
384	NR3C1
385	IL12B
386	FENDRR
387	SELP
388	HTR2A
389	LOC111674472
390	MYLK
391	STAT4
392	TLR5
393	MEN1
394	LITAF
395	AGER
396	ZAP70
397	PLG
398	FLT4
399	LRP5
400	CXCL12
401	HYDIN
402	HP
403	MIR107
404	MYBPC3
405	ECE1
406	PDGFB
407	NPHP3
408	SREBF1
409	CREBBP
410	HLA-G
411	PDE4A
412	C4A
413	ADAM17
414	HEXB
415	INVS
416	HSPA4
417	SRC
418	TNFSF11
419	COL3A1
420	G6PD
421	SLPI
422	EPHX1
423	NPHS2
424	HLA-C
425	AIRE
426	C12orf60
427	MAPK14
428	DES
429	TF
430	BCL2
431	PDCD1

432	C3
433	UCHL1
434	JUP
435	NOTCH2
436	HGF
437	BSCL2
438	CPS1
439	SLC9A3
440	RBP4
441	P2RX7
442	ENPP1
443	ATP2A2
444	SST
445	TARDBP
446	MIR203A
447	BMP2
448	CST3
449	CEACAM3
450	OFD1
451	TP63
452	MMP8
453	IL1R1
454	KCNN4
455	PMPCA
456	MT-CO3
457	CD40
458	OCRL
459	CSF2RB
460	NPHP1
461	GLB1
462	H2AC18
463	LDLR
464	SELE
465	LPL
466	MIR199A1
467	ALG9
468	SMN1
469	GC
470	CLCN6
471	CALR
472	CXCR2
473	HNRNPA1
474	NSD1
475	PLA2G7
476	MIR132
477	RARB
478	SOD2
479	FGA
480	CYP2D6
481	MIR144
482	EP300
483	MIR10A
484	CXCR1
485	MTM1

486	NTRK1
487	MT-ND5
488	GJB2
489	TG
490	GNAS
491	MT-CYB
492	MIR142
493	RPGR
494	RRM2B
495	MIR221
496	ICOSLG
497	TTC21B
498	MED13L
499	PTCH1
500	SYNJ1
501	CYCS
502	PROM1
503	LCAT
504	CD8A
505	CD28
506	TFRC
507	IFIH1
508	LOC111674475
509	TWNK
510	BMP4
511	WDR19
512	CYP1A1
513	SCARB2
514	ERCC6
515	PDE5A
516	TLR9
517	CD34
518	PRSS1
519	PTPRC
520	USH2A
521	CD80
522	CLN3
523	STX1A
524	TNFRSF1B
525	ABCB11
526	IL9
527	HSPB8
528	HMGB1
529	PAX2
530	IL12RB1
531	CBL
532	KNG1
533	ETV6
534	SP110
535	STAT5B
536	RETN
537	SHH
538	NOTCH3
539	IL33

540	JUN
541	MEG3
542	PLA2G6
543	CHRM3
544	CSF3R
545	INSL6
546	SMAD2
547	POLR1C
548	KCNQ1
549	KRT18
550	GUSB
551	IFT140
552	RAB7A
553	LCN2
554	SYK
555	SDHD
556	MIR125A
557	GATA2
558	CD14
559	NODAL
560	IFNGR1
561	PRKCSH
562	SETD2
563	NFKBIA
564	TSLP
565	HHIP
566	CHD7
567	STK11
568	SYP
569	MIR140
570	CEACAM6
571	CYP1B1
572	SLC25A4
573	GHRL
574	SEC63
575	RELA
576	HAMP
577	SCO2
578	FOXJ1
579	CUBN
580	MT-CO2
581	KL
582	TREM2
583	GCLC
584	MIR29C
585	IFNA2
586	CX3CR1
587	MMACHC
588	FLNC
589	CHRNA3
590	BTK
591	LOX
592	GZMB
593	SOD3

594	TAC1
595	HSPD1
596	TREX1
597	CCR3
598	BGLAP
599	MIR29B1
600	TLR3
601	CCDC65
602	CITED2
603	GLE1
604	MT-ND4
605	POMC
606	JAK3
607	IL12A
608	GSR
609	BLM
610	CYP27A1
611	MIR34C
612	NLRP1
613	DMD
614	SMC1A
615	FLT1
616	SH2B3
617	PHKA2
618	CDKN1A
619	KDR
620	IDS
621	YARS1
622	PFKM
623	SERPINH1
624	SGO1
625	FLG
626	GGT1
627	MED12
628	AHI1
629	LOC113664106
630	SMO
631	MIR181A2
632	DNAH11
633	IFT172
634	MIR424
635	TH
636	CCR4
637	MT-ND6
638	CAV3
639	F12
640	SCNN1A
641	GP1BA
642	MET
643	TBX20
644	TEK
645	CHI3L1
646	SMAD6
647	SOX9

648	KRT7
649	F13A1
650	SLC2A1
651	MIR210
652	PHOX2B
653	IL6R
654	APOH
655	SURF1
656	PRL
657	ADM
658	PRKCD
659	XIAP
660	OPTN
661	PAX6
662	CHGA
663	HDAC9
664	GATA1
665	HLA-DPA1
666	S100B
667	ENO2
668	NPHS1
669	MT-TK
670	CR2
671	DNAI1
672	CCL18
673	MIR122
674	CERS1
675	NPHP4
676	ADAMTS13
677	IL7R
678	POLR2F
679	CTSD
680	GSTM3
681	BAX
682	GYS1
683	CR1
684	MIR16-1
685	MYD88
686	RAD21
687	MYH9
688	GNPTAB
689	HSP90AA1
690	BTNL2
691	GM2A
692	ZEB2
693	LOC113633877
694	DZIP1L
695	LZTR1
696	TNFSF13B
697	IQCB1
698	ARSA
699	PRF1
700	NGF
701	CCDC40

702	IGF2
703	CD19
704	RAG1
705	NR4A2
706	MIR19A
707	IL3
708	ACHE
709	COL7A1
710	SOST
711	ACP5
712	LEPR
713	TTN-AS1
714	MB
715	F7
716	ANO5
717	CTSG
718	PDE4D
719	GRN
720	GRIN2B
721	ITGB3
722	TGFB3
723	CSF1
724	PRKAR1A
725	HDAC2
726	SGSH
727	NFE2L2
728	ATRX
729	ALK
730	SDHA
731	STRA6
732	PYGL
733	TPO
734	TYR
735	ITCH
736	IFNB1
737	GAPDH
738	FGF7
739	CREB1
740	LMOD1
741	NBN
742	FCGR3B
743	OPA1
744	PRKCQ
745	SELL
746	NRTN
747	HSPA1A
748	ARG1
749	FKRP
750	KITLG
751	ODAD3
752	TBK1
753	MIR483
754	NEK8
755	TMCO1

756	IGFBP3
757	DEFB1
758	LPA
759	F11
760	S100A9
761	GSTT1
762	FARSB
763	TINF2
764	SAG
765	MYOD1
766	NR3C2
767	ERCC2
768	FGF8
769	CFI
770	HBG2
771	GJC2
772	IL17RA
773	RNF213
774	TBX18
775	KMT2A
776	BEST1
777	U2AF1
778	ACTB
779	CCL4
780	COL11A1
781	CARD9
782	IRF4
783	RSPH4A
784	EBF3
785	VIP
786	DCTN4
787	IL2RB
788	FIP1L1
789	BBS10
790	SP140
791	ZFPM2
792	IRF1
793	FBLN5
794	IREB2
795	MDM2
796	PTH
797	CTNS
798	POMGNT1
799	CHEK2
800	DNAAF4
801	ADA2
802	HFE-AS1
803	SMARCA4
804	ADAR
805	IKBKG
806	SPARC
807	ANXA5
808	SCN4A
809	DKC1

810	PECAM1
811	POT1
812	IL15
813	S100A8
814	LOC111674477
815	MUSK
816	CAPN3
817	COL17A1
818	ADH1C
819	SLC6A14
820	ABCC2
821	AFP
822	CCL20
823	MIR148A
824	WAS
825	LGALS3
826	PTGS1
827	TCF4
828	ATP8B1
829	DICER1
830	ASAH1
831	TLR1
832	CACNA1A
833	FGB
834	PSTPIP1
835	IL4R
836	ODAD2
837	GSN
838	DNAH8
839	CD274
840	MVK
841	RPGRIP1L
842	TPP1
843	XK
844	HNMT
845	NAGA
846	RYR2
847	SLC26A9
848	TAB2
849	SMN2
850	PPARGC1A
851	DCTN1
852	ATG16L1
853	TRPV1
854	RAC2
855	ADAM33
856	CD86
857	NEK1
858	PSMB8
859	NOS1
860	CBS
861	PIK3CD
862	GALNS
863	CHIT1

864	KRT8
865	CXCL9
866	CCL17
867	HNF4A
868	AGTR2
869	WFS1
870	CXCL1
871	CDKN2B
872	CASP1
873	CCR1
874	KAT6B
875	MIR150
876	IL7
877	BRIP1
878	GLI3
879	SAA1
880	MIR30A
881	NF2
882	PIK3R1
883	NHP2
884	SDHC
885	HCRT
886	PITX2
887	F10
888	SIGLEC5
889	POSTN
890	SPG7
891	SLC6A3
892	PRPS1
893	NDE1
894	PLOD1
895	FMR1
896	PRKG1
897	ARSB
898	CETP
899	VPS35
900	MT-ND2
901	CYP2C9
902	PALB2
903	LMX1B
904	TSHR
905	SCNN1B
906	HBA1
907	SELENON
908	AHDC1
909	ITGA4
910	GIGYF2
911	TNFRSF13B
912	LTF
913	SERPINB1
914	MMP7
915	RSPH9
916	GRP
917	P2RY12

918	TAP1
919	DCDC2
920	KARS1
921	GLI2
922	BBS1
923	MIR93
924	MAPK3
925	BNC2
926	IGHE
927	FCGR3A
928	SCNN1G
929	CDH23
930	CCR7
931	ATP13A2
932	SFTA3
933	MCIDAS
934	BCHE
935	ABCA7
936	MSH2
937	KRT5
938	NR1H4
939	PEX6
940	HSPG2
941	BMP1
942	TRPC6
943	HNF1A
944	KCNH2
945	TLR7
946	TNFAIP3
947	NPY
948	ALPL
949	BCL2L1
950	CD27
951	DSTYK
952	DNMT3A
953	DNAI2
954	PROS1
955	CRH
956	ZIC3
957	TIMP2
958	MKKS
959	CLCN7
960	SAMHD1
961	CASP9
962	INSR
963	FOXC1
964	PTX3
965	SPG11
966	IGF1R
967	MIR148B
968	FLT3
969	ABCG5
970	SATB2
971	SIRT1

972	MITF
973	CD38
974	HNRNPA2B1
975	DNAL1
976	BCS1L
977	COL5A1
978	FLCN
979	CCDC39
980	DNAAF2
981	NEK9
982	CYP2E1
983	RUNX2
984	PPARA
985	RAPSN
986	KMT2D
987	ATP12A
988	PIK3C2A
989	TNFSF10
990	NCAM1
991	LBR
992	PRMT7
993	TAP2
994	CDKN1B
995	LIPC
996	GDF15
997	PPP1CB
998	NAT2
999	NRXN1
1000	NOP10
1001	TGM2
1002	VIM
1003	SLC22A4
1004	CFAP298
1005	PON2
1006	LRBA
1007	ERBB4
1008	DNAH9
1009	STAT6
1010	BMPR1A
1011	SCN1A
1012	SEMA3C
1013	MT-ND3
1014	MLH1
1015	SLC4A1
1016	ACE2
1017	ANGPT2
1018	HADHA
1019	ATP4A
1020	SH2D1A
1021	ITGB2
1022	NPM1
1023	PF4
1024	DNAAF11
1025	GNAQ

1026	TRAF3IP1
1027	RAC1
1028	GCH1
1029	MIRLET7D
1030	XDH
1031	TCIRG1
1032	DNMT1
1033	G6PC3
1034	A2ML1
1035	IRS1
1036	MECOM
1037	DNAAF1
1038	JAK1
1039	SLC18A3
1040	MT-ATP8
1041	RHO
1042	EFEMP2
1043	CLU
1044	ABCC8
1045	GRIN1
1046	NKX2-6
1047	CLCA4
1048	CD55
1049	GATA5
1050	DRC1
1051	SMARCAL1
1052	MCL1
1053	KCNJ11
1054	FTL
1055	DUOX2
1056	RAG2
1057	COX5A
1058	MT-TS1
1059	CIITA
1060	MIR27A
1061	EDAR
1062	ACTN4
1063	MIR222
1064	SLC22A5
1065	MAN2B1
1066	BMP7
1067	RNASEH2C
1068	GNB3
1069	TPM1
1070	DPYD
1071	ABCC6
1072	MIR98
1073	DNMT3B
1074	TNFSF15
1075	CD209
1076	CCR2
1077	MAPK8
1078	L1CAM
1079	LDHA

1080	MYL2
1081	LIG4
1082	DNAAF3
1083	ELOVL4
1084	CTRC
1085	TRB
1086	AARS2
1087	TGIF1
1088	BACH2
1089	CYP3A4
1090	SLC26A1
1091	MSH6
1092	IFNGR2
1093	MIR18A
1094	SLC26A3
1095	ODAD1
1096	CDC42
1097	MKI67
1098	MBP
1099	MIR181A1
1100	IKZF1
1101	CCL22
1102	MIR22
1103	NALCN
1104	DRD2
1105	CTSB
1106	ABCG8
1107	CFAP300
1108	NIPBL
1109	DNAH1
1110	HGSNAT
1111	GJA5
1112	SOCS1
1113	IKZF3
1114	ITGAL
1115	SPAG1
1116	CD81
1117	DNASE1
1118	BCL6
1119	MTR
1120	VEGFC
1121	HAVCR1
1122	TUG1
1123	ANKS6
1124	CCDC103
1125	H19
1126	PCSK9
1127	ZMYND10
1128	BMPR1B
1129	CFAP221
1130	AQP2
1131	DRD4
1132	ERCC1
1133	GHR

1134	GCG
1135	THBS1
1136	AFF4
1137	RNASEH2B
1138	GP1BB
1139	ZMPSTE24
1140	GSK3B
1141	TRAF3IP2
1142	CLDN2
1143	MIR195
1144	ACADVL
1145	CLN6
1146	POGZ
1147	TNFRSF10A
1148	UGT1A1
1149	EZH2
1150	MIR486-1
1151	CD44
1152	SERPINF2
1153	BAG3
1154	NME8
1155	PIK3CG
1156	C4B
1157	ALDH2
1158	CLCA1
1159	GNRH1
1160	AQP1
1161	PAH
1162	CYP19A1
1163	PMM2
1164	ODAD4
1165	MATR3
1166	CYP1A2
1167	DNAAF5
1168	NBAS
1169	CARD14
1170	TAMM41
1171	DOK7
1172	MUC7
1173	AVP
1174	TMEM216
1175	DDX58
1176	SMARCB1
1177	PACRG
1178	CLDN4
1179	COL18A1
1180	MMP13
1181	XRCC1
1182	HPRT1
1183	GDF2
1184	CRYAB
1185	EPAS1
1186	CCK
1187	LAMA2

1188	RSPH3
1189	STAT5A
1190	MT-TN
1191	BCL10
1192	CDKN1C
1193	PI3
1194	FOXG1
1195	CSTB
1196	MIR499A
1197	CD59
1198	PLA2G2A
1199	GAS8
1200	GPX1
1201	TTC12
1202	CCNO
1203	MYRF
1204	SOS2
1205	PLAUR
1206	MIR31
1207	ABCC9
1208	NEB
1209	NRG1
1210	NQO1
1211	MIR34B
1212	MBTPS2
1213	ADAM10
1214	CYP2C19
1215	LTBP2
1216	AREG
1217	STING1
1218	RSPH1
1219	HMGCR
1220	MYOC
1221	FOS
1222	POLG2
1223	ERAP1
1224	SERPINE2
1225	MIR200B
1226	ITGA2B
1227	EPCAM
1228	LRP1
1229	MIR141
1230	DNASE1L3
1231	PNPLA2
1232	GH1
1233	MMP14
1234	CASP10
1235	YAP1
1236	LOC111674463
1237	CXCL5
1238	NOX4
1239	RPS27A
1240	RBCK1
1241	LMNB1

1242	FOXE3
1243	GUCY1A1
1244	TMEM231
1245	PARP1
1246	RB1
1247	IL37
1248	PTH1R
1249	CRKL
1250	PPIG
1251	FHIT
1252	EMD
1253	IDH1
1254	CRB2
1255	MLXIPL
1256	NDUFS4
1257	GREM1
1258	TGM1
1259	CDK4
1260	CSF1R
1261	STK36
1262	HAVCR2
1263	CD69
1264	WRAP53
1265	LRP2
1266	CLCN1
1267	CYP27B1
1268	DNAAF6
1269	RPGRIP1
1270	RAD51
1271	BAP1
1272	CARMIL2
1273	PGR
1274	MIR25
1275	CDH2
1276	LAMB2
1277	NFU1
1278	SPEF2
1279	HDAC8
1280	MIR214
1281	MS4A1
1282	IGFBP1
1283	LIFR
1284	VPS33A
1285	SHOC2
1286	IRGM
1287	SLC12A3
1288	MIR152
1289	CLN5
1290	WIPF1
1291	IGF2R
1292	NEU1
1293	MYCN
1294	RHOA
1295	ARX

1296	IFT80
1297	PKD1L1
1298	SYNGAP1
1299	ASCL1
1300	PLEC
1301	RNU4ATAC
1302	PEX1
1303	ITGA3
1304	FGF20
1305	RAI1
1306	GDF6
1307	CHRNG
1308	SCT
1309	GNE
1310	CDK5
1311	POFUT1
1312	MIR28
1313	SNAI2
1314	IL16
1315	SON
1316	UFD1
1317	APOC3
1318	CCR8
1319	FKTN
1320	PURA
1321	CACNA1C
1322	ITPR1
1323	HSPA5
1324	TLL1
1325	SGO1-AS1
1326	SERPINF1
1327	FXN
1328	IL11
1329	MIR9-1
1330	LRP6
1331	C11orf65
1332	CRYAA
1333	ITGAX
1334	MIR192
1335	BIRC5
1336	PKP2
1337	STXBP2
1338	RASA2
1339	GTF2I
1340	GYG1
1341	NEK10
1342	DNAJC5
1343	NDUFS2
1344	GAS2L2
1345	FBN2
1346	ARID1B
1347	TNFRSF8
1348	CTSK
1349	NR5A1

1350	TFAP2A
1351	DPP4
1352	COL11A2
1353	SEMA3D
1354	MIR130A
1355	ACTN2
1356	RBPJ
1357	TBX21
1358	CDKN2B-AS1
1359	DNAH7
1360	SLC30A10
1361	PRODH
1362	SLC26A4
1363	GAST
1364	HIRA
1365	WRN
1366	MALAT1
1367	SDC1
1368	LAMA3
1369	MSR1
1370	PCNA
1371	RNASEH2A
1372	RASA1
1373	MT-TL2
1374	DGUOK
1375	VANGL1
1376	SEC61A1
1377	OPRM1
1378	STXBP1
1379	SLC2A10
1380	ELP4
1381	MT-TF
1382	IL22
1383	WNT1
1384	TYK2
1385	CALB2
1386	PPBP
1387	TCAP
1388	UBE3A
1389	CEACAM5
1390	KDM6A
1391	FHL1
1392	SLC29A3
1393	WDR35
1394	SLC40A1
1395	SMAD7
1396	C5
1397	CDKN3
1398	PROC
1399	CHRNA1
1400	SP1
1401	CD2AP
1402	KRT19
1403	EPX

1404	EVC2
1405	MIR30E
1406	DDX3X
1407	LIMK1
1408	LDB3
1409	SLC7A7
1410	TTC7A
1411	RRAS
1412	MIR200A
1413	IL6ST
1414	FLI1
1415	ALAD
1416	ANKRD1
1417	DNAJB13
1418	HMGA2
1419	WNT5A
1420	RAB9B
1421	VCL
1422	MT-ND4L
1423	SLC12A1
1424	CHUK
1425	DLL4
1426	MTRR
1427	MAP3K7
1428	ANGPT1
1429	IDO1
1430	SCGB3A2
1431	ACAN
1432	CAMP
1433	MYOCD
1434	LOC107988032
1435	DEFB4A
1436	CYP11B2
1437	NPPC
1438	GTF2IRD2
1439	IL23A
1440	ACVR1
1441	IRF3
1442	MEF2C
1443	EWSR1
1444	MIR205
1445	MICA
1446	PPT1
1447	ALPP
1448	GNS
1449	PEPD
1450	SEC24C
1451	CEP164
1452	EIF2AK2
1453	POGLUT1
1454	F2RL1
1455	AQP5
1456	IDH2
1457	MYO5A

1458	AHSG
1459	SERPINA6
1460	MIR133B
1461	BBS2
1462	TRPM7
1463	SKIV2L
1464	PGF
1465	SCN1B
1466	VKORC1
1467	S100A12
1468	NR2E3
1469	CYP3A5
1470	MYO5B
1471	MAGEL2
1472	MASP2
1473	DBH
1474	FANCC
1475	IL2RG
1476	CXCL2
1477	CTCF
1478	SOCS3
1479	APAF1
1480	SPTAN1
1481	PDX1
1482	EYA1
1483	SNRPN
1484	STX3
1485	AKT2
1486	SLCO2A1
1487	OCA2
1488	TK2
1489	TKT
1490	TFAM
1491	PIGL
1492	SYNE1
1493	NUP107
1494	BBS12
1495	RIT1
1496	SPAST
1497	MIRLET7B
1498	GCK
1499	AVPR2
1500	LAMP1
1501	MIR196A1
1502	RRAS2
1503	ACTG1
1504	SMC3
1505	ATXN8OS
1506	EHMT1
1507	TBCE
1508	APOA5
1509	POLD1
1510	CYP2A6
1511	PTGIS

1512	DMPK
1513	KLRK1
1514	OGG1
1515	CDK2
1516	HTRA1
1517	KIAA0586
1518	PRKACA
1519	UTS2
1520	CEP83
1521	TBX2
1522	PTPN2
1523	KRT20
1524	COL9A3
1525	FLNB
1526	ALDH18A1
1527	ICOS
1528	PTHLH
1529	ATXN7
1530	CFP
1531	IFNL3
1532	CACNA1F
1533	PRDM16
1534	KLK3
1535	WWOX
1536	TCOF1
1537	COQ8B
1538	SOX2
1539	HSPA8
1540	KEAP1
1541	DDC
1542	ARVCF
1543	TGFA
1544	ITGA8
1545	BBS4
1546	TLR6
1547	RREB1
1548	FZD4
1549	PML
1550	DKK1
1551	MIR96
1552	CD5
1553	CS
1554	LEPQTL1
1555	MIR342
1556	KIR3DL1
1557	MAP2K2
1558	PDGFA
1559	HTR1B
1560	DOCK8
1561	ANKRD11
1562	AGRN
1563	TBCK
1564	BECN1
1565	PRSS2

1566	NTRK2
1567	CA2
1568	CX3CL1
1569	PDPN
1570	FANCD2
1571	CDK8
1572	CHRNA5
1573	ADRB1
1574	TPM3
1575	ESR2
1576	NAMPT
1577	FLNC-AS1
1578	BIRC3
1579	GYPA
1580	APOA2
1581	ADCY10
1582	YY1AP1
1583	AQP4
1584	ROR2
1585	TPH1
1586	SGCB
1587	PHEX
1588	CCND2
1589	TREM1
1590	ERF
1591	MIR338
1592	SLC19A1
1593	PAX3
1594	KANSL1
1595	FOXO3
1596	HTR1A
1597	IL1RAPL2
1598	HSPA1L
1599	NTS
1600	BGN
1601	CYP17A1
1602	CSPP1
1603	MRC1
1604	MIR125B1
1605	LACTB
1606	MIR127
1607	TIMP3
1608	OTC
1609	KCNA5
1610	SLC25A13
1611	FKBP10
1612	APEX1
1613	FANCM
1614	LACC1
1615	BLK
1616	ABCC1
1617	MAOB
1618	EPOR
1619	MUC16

1620	MIR200C
1621	ANKH
1622	MIR24-1
1623	USB1
1624	ANG
1625	RARA
1626	PLA2G4A
1627	CNR1
1628	SALL1
1629	TNFRSF10B
1630	SLC20A2
1631	SUFU
1632	DNM1L
1633	CHRNA4
1634	FCER2
1635	POMT1
1636	FANCI
1637	FH
1638	ANXA11
1639	NTRK3
1640	NOX1
1641	TXN
1642	GRK2
1643	ATF6
1644	ETS1
1645	IRAK1
1646	SLC12A2
1647	DLL1
1648	CDK1
1649	TPMT
1650	FBXW7
1651	SHBG
1652	MRAS
1653	KCNJ5
1654	MIR185
1655	ENTPD1
1656	TMEM43
1657	MT-TH
1658	TRPM4
1659	GH-LCR
1660	HNRNPK
1661	TMEM237
1662	MEF2A
1663	GP6
1664	ATP1A3
1665	PRKCA
1666	FABP3
1667	TCTN2
1668	ATP1A1
1669	SMARCA2
1670	SLC12A6
1671	SLC10A2
1672	FKBP6
1673	CCNA2

1674	ALG8
1675	HRH2
1676	ALDOA
1677	IFT88
1678	PRKD1
1679	DCN
1680	PIGN
1681	IKBKB
1682	CD163
1683	TPM2
1684	TNFSF12
1685	HOTAIR
1686	MT-TT
1687	MIR23B
1688	EIF2AK3
1689	KAT6A
1690	MIR197
1691	TMEM126B
1692	CLIP2
1693	JMJD1C
1694	DNAJC30
1695	SLC18A2
1696	LTBP3
1697	BTD
1698	C1S
1699	PAX5
1700	HRH1
1701	ASS1
1702	SETD5
1703	CEP120
1704	PDHA1
1705	DHCR7
1706	UBE2L3
1707	NR1H3
1708	MT-TI
1709	MYB
1710	MUC2
1711	LYN
1712	MUTYH
1713	MIR92A1
1714	KCNMA1
1715	EPHB4
1716	SLC34A3
1717	MUCL3
1718	MST1
1719	PIEZO1
1720	FREM2
1721	MHRT
1722	KIAA1109
1723	OSM
1724	KLRC4
1725	EGR1
1726	FAT4
1727	CLEC1A

1728	AXL
1729	SUMF1
1730	PAFAH1B1
1731	CLDN3
1732	TCTN3
1733	NTF3
1734	IVNS1ABP
1735	ADORA2A
1736	FANCE
1737	BAK1
1738	FGF1
1739	SYT2
1740	COL5A2
1741	PKLR
1742	NEAT1
1743	STUB1
1744	DAG1
1745	NFKBIL1
1746	TTC25
1747	PIH1D3
1748	CCDC151
1749	CCDC114
1750	ARMC4
1751	LRRC6
1752	GLP1R
1753	BMF
1754	FHL5
1755	CD47
1756	GPR68
1757	MAGT1
1758	ACTBL2
1759	ALPI
1760	A2M
1761	DNAH14
1762	LCN1
1763	POTEM
1764	IAPP
1765	FEV
1766	P2RX3
1767	POTEKP
1768	LTC4S
1769	ACOT7
1770	ABCG2
1771	ADORA1
1772	ADORA2B
1773	ADRB3
1774	ANXA1
1775	CES1
1776	CHRM1
1777	CHRM2
1778	CHRM4
1779	CHRM5
1780	CPNE1
1781	CYP2B6

1782	CYP2C18
1783	CYP2C8
1784	CYP3A43
1785	CYP3A7
1786	ftsI
1787	GLT6D1
1788	GLTP
1789	HM13
1790	KCNK9
1791	mrcA
1792	mrcB
1793	mrdA
1794	nedA
1795	NOMO1
1796	ORM1
1797	ORM2
1798	PDE2A
1799	PDE3A
1800	PDE4B
1801	PDE4C
1802	RIC3
1803	SLC15A1
1804	SLC15A2
1805	SLC22A1
1806	SLC22A2
1807	SLC22A3
1808	SLC22A6
1809	SLC22A7
1810	SLC22A8
1811	SLC47A1
1812	SLCO1A2
1813	SLCO1B1
1814	SLCO1B3
1815	SULT1A1
1816	SULT1A2
1817	SULT1A3
1818	SULT1A4
1819	SULT1B1
1820	SULT1C2
1821	SULT1C3
1822	SULT1C4
1823	SULT2A1
1824	SULT4A1
1825	SULT6B1
1826	TTLL3
1827	UGT1A8
1828	UGT1A9
1829	UGT2B15
1830	UGT2B7
1831	xlnA
